# A broad-spectrum antiviral molecule, QL47, selectively inhibits eukaryotic translation

**DOI:** 10.1074/jbc.RA119.011132

**Published:** 2019-12-30

**Authors:** Mélissanne de Wispelaere, Margot Carocci, Dominique J. Burri, William J. Neidermyer, Calla M. Olson, Imme Roggenbach, Yanke Liang, Jinhua Wang, Sean P. J. Whelan, Nathanael S. Gray, Priscilla L. Yang

**Affiliations:** ‡ Department of Microbiology and Blavatnik Institute, Harvard Medical School, Boston, Massachusetts 02115; § Department of Biological Chemistry and Molecular Pharmacology, Harvard Medical School, Boston, Massachusetts 02115; ¶ Department of Cancer Biology, Dana-Farber Cancer Institute, Boston, Massachusetts 02215

**Keywords:** translation, small molecule, inhibition mechanism, antiviral agent, anticancer drug

## Abstract

Small-molecule inhibitors of translation are critical tools to study the molecular mechanisms of protein synthesis. In this study, we sought to characterize how QL47, a host-targeted, small-molecule antiviral agent, inhibits steady-state viral protein expression. We demonstrate that this small molecule broadly inhibits both viral and host protein synthesis and targets a translation step specific to eukaryotic cells. We show that QL47 inhibits protein neosynthesis initiated by both canonical cap-driven and noncanonical initiation strategies, most likely by targeting an early step in translation elongation. Our findings thus establish QL47 as a new small-molecule inhibitor that can be utilized to probe the eukaryotic translation machinery and that can be further developed as a new therapeutic agent.

## Introduction

Cellular translation is a finely regulated biosynthetic reaction that yields proteins, the final products of gene expression. Small molecules that can tune protein synthesis have been essential in studying the translation machinery. The ribosome in particular is the main target of a multitude of antibacterial agents that inhibit translation, and these small molecules have served as powerful tools to interrogate the functions of the prokaryotic ribosome (1). Although there is a remarkable degree of conservation between prokaryotic and eukaryotic translation, there are major differences in the composition and function of their ribosomes (2). This has been particularly highlighted by the discovery of multiple inhibitors that specifically target only prokaryotic or eukaryotic translation. Although far fewer eukaryote-specific ribosomal inhibitors have been identified, understanding their molecular mechanisms is of particular interest, as it can highlight some major functional differences between those translation machineries.

Because protein synthesis is tightly coupled to cell growth and function, small molecules that dysregulate translation are also of interest to treat diseases, as exemplified by the historical example of antibiotics that inhibit prokaryotic translation (1). Tumor development is often driven by dysregulation of translation, and small molecules that target the eukaryotic ribosome have the potential to restrain cancer cell growth (3). Viral pathogens are also absolutely dependent on the host translation machinery to establish an infectious cycle, and multiple small-molecule inhibitors of cellular translation have been shown to exert antiviral activity (4, 5).

We previously reported an approach to target the cellular reactive cysteinome (6) to identify inhibitors of host factors required for the infectious cycle of dengue virus (DV),^4^ a mosquito-borne flavivirus that is a significant pathogen in tropical regions (7). This effort led to the discovery of multiple antiviral compounds (6), including QL47, a cysteine-reactive small molecule that inhibits the proliferation of Bruton's tyrosine kinase (BTK)–dependent cancer cell lines (8). We demonstrated that QL47 inhibits not only flaviviruses but also several unrelated, medically relevant viruses, including poliovirus, Ebola virus, and human orthopneumovirus (6, 9, 10), consistent with a host- rather than virus-targeting mode of action. Subsequent structure–activity relationship (SAR) studies revealed that QL47's antiviral activity is closely associated with inhibition of steady-state viral protein expression (6, 9). We demonstrated that TEC-family kinases such as BMX or BTK, the previously characterized targets of QL47 (8), are not responsible for antiviral activity (9). This finding, along with the observation that BMX-IN-1, an analog of QL47 equipotent at inhibiting BTK, exhibits less potent antiproliferative activity (8) and less antiviral activity (9) in cells that do not express BMX, suggest that QL47 has additional cellular targets that contribute to these activities.

In this study, we sought to investigate the mechanism(s) responsible for QL47's effect on steady-state viral protein expression. The DV genome is a positive-sense, single-stranded RNA that is translated as a single ORF. The polyprotein produced is co- and posttranslationally processed by both the viral NS2B/3 protease and cellular peptidases. Translation of the DV genome following viral entry into the host cell is exquisitely dependent on the host translational machinery, as DV and other viruses do not package or encode their own translation factors. The genome of DV has a 7-methyl guanosine cap structure, and although it is not polyadenylated, its 3′ UTR contributes to efficient translation in a manner comparable with cellular poly(A) tails (11–13). Although it is generally accepted that DV is translated via canonical cap-dependent translation, DV has also been shown to be translated via an alternative eIF4E-independent pathway (14, 15) and to harbor an internal ribosomal entry site in its 5′ UTR (16). There is currently little understanding of the significance of these alternate translation pathways within the DV infectious cycle. More generally, the strategies employed by DV to regulate host and viral translation are still poorly understood. Thus, the identification of small-molecule inhibitors that impact DV translation provides us with pharmacological tools to interrogate this viral process.

Here we show that QL47 is a general inhibitor of translation and demonstrate that this activity is specific to the eukaryotic translation machinery. We additionally provide evidence that QL47 acts at an early stage in protein synthesis and can exert its inhibition regardless of the translation initiation strategy used, host or viral. The finding that multiple viral pathogens are sensitive to QL47 activity suggests that viruses could employ a specific host pathway for translation of their genomes that can be targeted with small molecules. These findings suggest that QL47 is a multitargeted small molecule that can target specific kinases such as BTK and BMX (8) but can also target additional unknown cellular factors that are important for viral translation.

## Results

### QL47 inhibits viral and host protein synthesis

**Figure 1.**
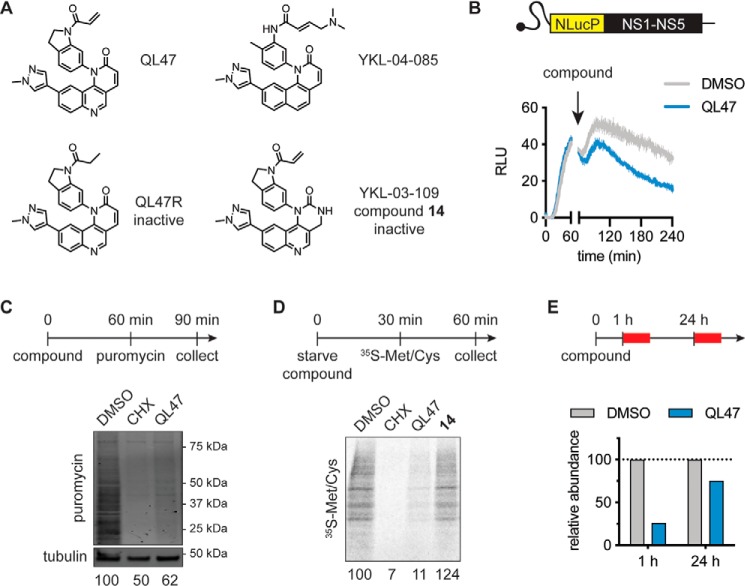
**QL47 inhibits protein synthesis in live cells.** *A*, chemical structures of QL47 and the derivatives YKL-04-085, QL47R, and YKL-03-109 (compound 14) (6, 9). QL47R is an inactive analog due to replacement of the cysteine-reactive acrylamide moiety with a nonreactive propyl amide. Compound 14 is a negative control compound with significantly diminished antiviral activity. *B*, HEK293T cells were transfected with an *in vitro* transcribed reporter DV subgenomic RNA bearing the virus's seven nonstructural genes (*NS1–NS5*) as well as a NanoLuc®-proline, glutamate, serine, threonine (*NlucP*) luciferase reporter gene. The intracellular reporter activity was immediately measured for 1 h at 37 °C. Cells were treated with DMSO or 2 μm QL47 at 1 h post-transfection, and the intracellular luciferase signal was then measured continuously for 3 h at 37 °C. The luciferase signal obtained from mock-transfected cells was subtracted, and data are presented as means ± S.D. of three experimental replicates. One representative experiment is shown from two independent experiments. *RLU*, relative light units. *C*, measurement of puromycin incorporation in nascent cellular proteins. Huh7 cells were treated with DMSO, 50 μg/ml CHX, or 2 μm QL47 for 1 h. The cells were next pulse-labeled with 1 μm puromycin for 30 min, and their cellular contents were analyzed by Western blotting. Prematurely terminated polypeptides were detected using a puromycin-specific antibody. Their abundance was normalized to the loading control (tubulin) and is presented as a percentage of the DMSO-treated control samples. One representative experiment is shown from two independent experiments. *D*, metabolic labeling of cellular proteins with radiolabeled amino acids. Huh7 cells were starved for 30 min in methionine/cysteine-free medium and concomitantly treated with DMSO, 30 μg/ml CHX, 2 μm QL47, or 2 μm compound 14. The medium was next supplemented with a mixture of ^35^S-Met and ^35^S-Cys for 30 min. Total cell lysates were analyzed by SDS-PAGE, followed by autoradiography to measure bulk protein synthesis. Neosynthesized protein abundance is presented as a percentage of the DMSO-treated control samples. One representative experiment is shown from three independent experiments. *E*, Huh7 cells were treated with DMSO or 2 μm QL47, and metabolic labeling (*red boxes*) was performed as indicated in *D* 1 h and 24 h post-treatment. Analysis of the neosynthesized proteins was performed as in *D*, and their abundance is presented as a percentage of the DMSO-treated control samples at each time point. One representative experiment is shown from two independent experiments.

Our prior SAR studies yielded multiple compounds that revealed the chemical features necessary for QL47's inhibition of steady-state viral protein expression (9). We demonstrated that covalent modification of QL47 targets is essential, as replacement of the cysteine-reactive acrylamide moiety with a nonreactive propyl amide yields an inactive compound, QL47R (Fig. 1*A*) (6, 9). We also identified acrylamide-containing derivatives, such as YKL-04-085 (Fig. 1*A*), that are not covalent inhibitors of TEC-family kinases targeted by QL47 (8, 9). YKL-03-109 (compound 14) is a structurally related analog with greatly reduced antiviral activity (Fig. 1*A*) (6, 9).

We demonstrated previously that QL47 and YKL-04-085, but not QL47R or compound 14, reduce luciferase reporter activity expressed in the context of authentic DV translation (6, 9). Because we have shown that QL47's antiviral effects cannot be explained by an effect on viral RNA abundance (6), these experiments indicated that QL47 reduces the steady-state abundance of proteins expressed from the viral subgenomic RNA in cells by affecting protein synthesis and/or turnover. To monitor the acute effects of QL47 on steady-state protein expression in cells, we designed a DV replicon in which a NanoLuc luciferase reporter protein is fused to a degradation sequence that reduces its intracellular half-life to 20 min (17) (Fig. 1*B*). Using this construct, we observed that addition of QL47 results in a rapid decrease in luciferase activity, consistent with reduced translation of the viral subgenomic RNA and/or increased turnover of the translated products in mammalian cells (Fig. 1*B*).

QL47's broad-spectrum antiviral activity and the dependence of most viruses on the host translational machinery prompted us to examine QL47's effect on translation. We performed metabolic labeling experiments and measured the incorporation of puromycin (Fig. 1*C*) or radiolabeled amino acids (Fig. 1*D*) into newly synthesized host proteins following 1-h live cell treatment. Consistent with a direct effect on RNA translation, we observed that QL47 prevents accumulation of newly synthesized host proteins in both assays. We also showed that compound 14 has no effect on translation in this assay (Fig. 1*D*). These observations were in agreement with our finding that QL47 could inhibit expression of *in vitro* transcribed reporter RNA that had the characteristics of cellular mRNA and harbored a cap structure and a poly(A) tail (Fig. S2). Interestingly, we observed that this effect on host protein synthesis is acute and lost when cells are treated with QL47 for 24 h (Fig. 1*E*). The fact that QL47's inhibition of protein accumulation is transient may also explain why this compound does not exhibit significant cellular cytotoxicity at the concentrations used in our assays (6). Collectively, these experiments demonstrate that QL47 has a direct effect on translation of both host and viral RNA.

### QL47 inhibits protein synthesis in vitro

**Figure 2.**
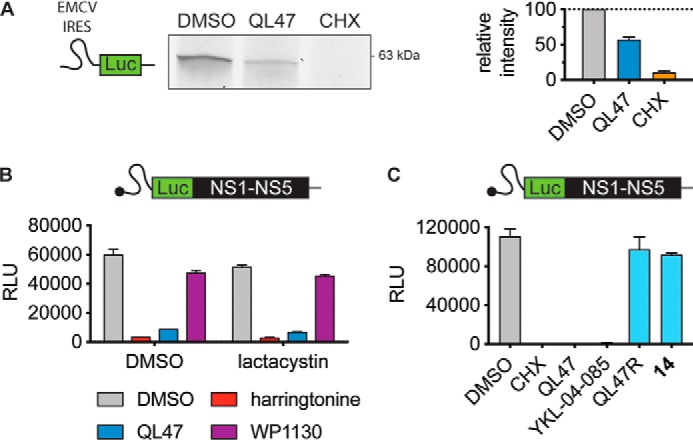
**QL47 inhibits protein synthesis *in vitro*.** *A*, SDS-PAGE analysis of *in vitro* translations performed in rabbit reticulocyte lysates for 90 min at 30 °C in the presence of precharged FluoroTect^TM^ lysine tRNA and DMSO, 40 μm QL47, or 30 μg/ml CHX. An *in vitro* transcribed reporter RNA bearing the EMCV IRES and a luciferase (*Luc*) reporter gene (42) was used as a template. One representative experiment is shown from three independent experiments. The abundance of neosynthesized fluorescent proteins was measured and is graphically presented on the *right*. Data are presented as means normalized to DMSO ± S.D. of two independent experiments. *B*, rabbit reticulocyte lysates were pretreated with DMSO or 25 μm proteasome inhibitor lactacystin for 15 min at room temperature. Subsequently, an *in vitro* transcribed reporter DV subgenomic RNA (40) was added, and lysates were incubated in the presence of DMSO or 40 μm of the indicated small molecules for 90 min at 30 °C. The luciferase signal was measured, and data are presented as means ± S.D. of two technical replicates. One representative experiment is shown from two independent experiments. *RLU*, relative light units. *C*, translation of *in vitro* transcribed reporter DV subgenomic RNA in rabbit reticulocyte lysates was performed for 90 min at 30 °C in the presence of DMSO, 30 μg/ml CHX, or 40 μm of QL47 and the indicated analogs. The luciferase signal was measured, and data are presented as means ± S.D. of two technical replicates. One representative experiment is shown from three independent experiments.

To examine QL47's effects on translation more directly, we next evaluated its activity in a cell-free protein synthesis system. In experiments paralleling the metabolic labeling assays performed on live cells (Fig. 1, *C* and *D*), we utilized a fluorescently labeled amino acid to monitor the accumulation of newly synthesized proteins in rabbit reticulocyte lysates. QL47 reduces the abundance of a luciferase reporter protein translated from the encephalomyocarditis virus (EMCV) internal ribosomal entry site (IRES) in this system (Fig. 2*A*), consistent with its effect on intracellular reporter protein abundance (6). We noted that the concentration of QL47 required to achieve significant inhibition of protein synthesis in rabbit reticulocytes lysates was significantly higher than in mammalian cells, which is likely due to the increased abundance of the target mediating this effect in these lysates (Fig. S1). Although this result strongly suggests a direct effect on translation, we could not exclude an effect of the compound on protein stability because of the presence of a functional proteasome in rabbit reticulocyte lysates (18, 19). To resolve this issue, we preincubated the lysates with the proteasome inhibitor lactacystin prior to treatment with QL47 or harringtonine, a well-characterized inhibitor of translation (20–22). As shown in Fig. 2*B*, lactacystin does not restore the abundance of newly translated protein in the *in vitro* translation reactions conducted in the presence of QL47 or harringtonine. Likewise, we detected limited impact by WP1130, a promiscuous deubiquitinase inhibitor that potently promotes protein degradation in cell-based assays (23), on the abundance of the subgenomic viral polyprotein in the *in vitro* translation system (Fig. 2*B*). Together, these results suggest that QL47's activity in the cell-free protein synthesis system is due to inhibition of RNA translation rather than enhancement of protein instability and/or induction of protein degradation.

To further demonstrate that this inhibition of *in vitro* translation is pertinent to QL47's cellular activity, we took advantage of our previously reported SAR studies (6, 9) and tested the activity of QL47 analogs in this system. Consistent with our hypothesis, we found a good correlation between their reported activities *in cellulo* and their activities in the *in vitro* translation assay (Fig. 2*C*). The active analog YKL-04-085 (9) showed potent inhibition of reporter protein synthesis *in vitro*, whereas the inactive compound 14 (6, 9) and the negative control compound QL47R (Fig. 1*A*) exhibited no significant inhibitory activity in this assay (Fig. 2*C*).

### QL47 does not inhibit prokaryotic protein synthesis

**Figure 3.**
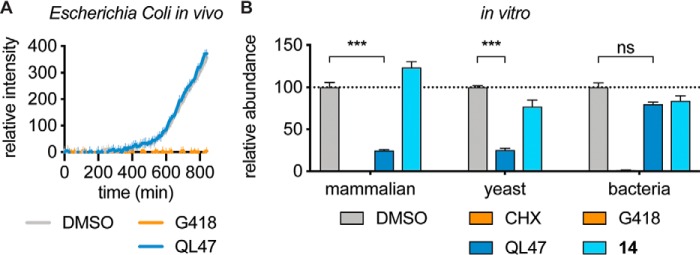
**QL47 inhibits eukaryotic but not prokaryotic protein synthesis.** *A*, *E. coli* cells carrying the pUA66-*rrnB* plasmid that constitutively expresses GFP (24) were treated with DMSO, 250 μg/ml G418, or 50 μm QL47. The intracellular GFP fluorescence signal was then measured continuously for 14 h at 37 °C. The signal obtained from growth medium was subtracted, and data are presented as means ± S.D. of 12 experimental replicates. One representative experiment is shown from two independent experiments. *B*, analysis of *in vitro* translation assays performed in rabbit reticulocyte lysates, yeast cell lysates, or a reconstituted *E. coli* cell-free synthesis system (PURExpress®). Translation in rabbit reticulocyte lysates was performed in the presence of DMSO, 30 μg/ml CHX, 40 μm QL47, or 40 μm compound 14. An *in vitro* transcribed reporter DV subgenomic RNA was used as a template, and the luciferase signal was measured after 90-min incubation at 30 °C. Data are presented as means normalized to DMSO ± S.D. of four experimental replicates. Translation in yeast cell lysates was performed in the presence of DMSO, 40 μm QL47, or 40 μm compound 14. An *in vitro* transcribed vesicular stomatitis virus (VSV) RNA bearing a luciferase reporter gene (44) was used as a template, and the luciferase signal was measured after 2-h incubation at 25 °C. Data are presented as means normalized to DMSO ± S.D. of three experimental replicates. Translation in a reconstituted *E. coli* cell-free synthesis system (PURExpress®) was performed in the presence of DMSO, 250 μg/ml G418, 100 μm QL47, or 100 μm compound 14. A plasmid expressing GFP under control of a T7 promoter was used as a template. After 1-h incubation at 37 °C, the total protein content was analyzed by Western blotting. The reporter protein was detected using a GFP antibody, and its abundance was normalized to the loading control (histidine tag). Data are presented as means normalized to DMSO ± S.D. of two technical replicates. One representative experiment is shown from four (rabbit reticulocyte lysates) or two (yeast cell lysates and *E. coli* cell-free synthesis system) independent experiments. *Asterisks* indicate that the differences between experimental samples and the DMSO-treated control samples are statistically significant when compared using unpaired *t* test: ***, *p* < 0.001; nonsignificant (*ns*), *p* > 0.05.

We have shown previously that QL47 exhibits antiviral activity in multiple mammalian cell lines and in insect cells (6), which suggests that QL47 broadly inhibits eukaryotic translation. Due to a high degree of conservation between eukaryotic and prokaryotic translation machineries (2), we asked whether QL47 could also inhibit prokaryotic translation. To examine this, we treated *Escherichia coli*, a model prokaryote, that was engineered to constitutively express a reporter GFP protein (24) with QL47, and monitored the GFP signal as a measure of steady-state protein expression and, hence, translation over time. Interestingly, we observed that QL47 did not inhibit bacterial protein synthesis in this system (Fig. 3*A*). Since this absence of inhibition could be attributed to a lack of cell permeability, we also showed that QL47 does not inhibit prokaryotic translation *in vitro* using a reconstituted cell-free synthesis system (25). QL47 does, however, inhibit translation in yeast cell lysates, demonstrating that this small molecule specifically affects eukaryotic translation (Fig. 3*B*).

### QL47 inhibits an early step in the translation process

**Figure 4.**
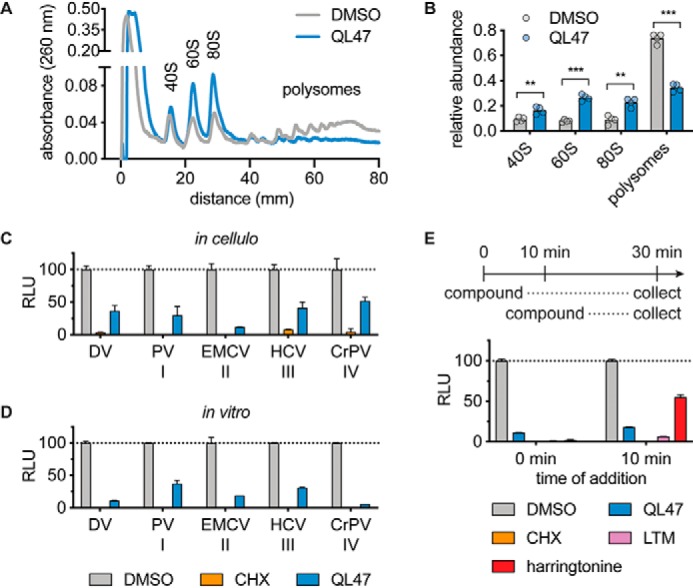
**QL47 inhibits an early step in the translation process.** *A*, polysome profiling. Huh7 cells were treated with DMSO or 2 μm QL47 for 3 h. Cell extracts were loaded on a sucrose density gradient and separated by ultracentrifugation. Sucrose gradients were eluted from the top using a fractionator, and RNA absorption at 260 nm was continuously recorded. Actively translated polysomal mRNAs were separated from mRNAs associated with the 80S monosome or single ribosomal units (40S and 60S). One representative experiment is shown from five independent experiments. *B*, quantification of polysomes and ribosomal units obtained in the polysome profiling experiments shown in *A*. The area under the curve for each peak was quantified, and data are presented as means normalized to the sum of all peaks area ± S.D. of four independent experiments. *Asterisks* indicate that the differences between experimental samples and the DMSO-treated control samples are statistically significant when compared using unpaired *t* test: ***, *p* < 0.001; **, *p* < 0.01. *C*, analysis of translation assays performed in live cells. Huh7 cells were transfected with various *in vitro* transcribed reporter RNAs. Reporter luciferase translation was driven by either the DV 5′ UTR, poliovirus type I IRES (41), EMCV type II IRES, HCV type III IRES (43), or CrPV type IV IRES. Cells were immediately treated with DMSO, 30 μg/ml CHX, or 2 μm QL47. The intracellular luciferase signal was measured 6 h post-treatment, and data are presented as means normalized to DMSO ± S.D. of two experimental replicates. One representative experiment is shown from four independent experiments. *RLU*, relative light units. *D*, analysis of *in vitro* translation assays performed in rabbit reticulocyte lysates. The *in vitro* transcribed reporter RNAs presented in *B* were used as templates. Translation was performed in the presence of DMSO, 30 μg/ml CHX, or 40 μm QL47. The reactions were incubated for 90 min at 30 °C, and the luciferase signal was measured. Data are presented as means normalized to DMSO ± S.D. of two technical replicates. One representative experiment is shown from two independent experiments. *E*, *in vitro* translations were performed in rabbit reticulocyte lysates using *in vitro* transcribed reporter DV subgenomic RNA as a template. Compounds were added either immediately after the translation reaction was assembled or after 10-min incubation at 30 °C. Samples were treated with DMSO, 40 μm QL47, 30 μg/ml CHX, 40 μm LTM, or 40 μm harringtonine. Data are presented as means normalized to DMSO values obtained for each treatment ± S.D. of two technical replicates. One representative experiment is shown from two independent experiments.

We next sought to examine the mechanism by which QL47 inhibits protein synthesis by analyzing active translation complexes in mammalian cells treated with the compound. Cell lysates were collected after a 3-h treatment and applied to a sucrose density gradient to separate the RNAs based on the number of ribosomes currently bound to that RNA. Each fraction was measured for its RNA content. Polysome profiles show that QL47 disrupts polysomes and promotes an increase in the single ribosomal units (40S, 60S) and the monosomal complex (80S) (Figs. 4, *A* and *B*). This polysome profile is reminiscent of those generated by inhibitors that block ribosomes at sites of translation initiation and allow the ribosomes to run off the RNA template (3, 21, 26–28). An overaccumulation in 80S ribosomes also suggests that QL47 blocks either a late step in initiation or an early step in elongation.

In an effort to determine whether QL47 targets a translation initiation factor, we took advantage of the diverse translation initiation strategies utilized by RNA viruses. Although flaviviruses, including DV, have a 5′ cap and initiate translation primarily via a canonical mechanism (11, 14, 16), many other RNA viruses have evolved strategies to initiate translation via mechanisms that do not require all factors of the canonical pathway. Notably, many viruses utilize IRES for direct recruitment of host ribosomes and eIFs to viral RNA and initiation of translation via cap-independent mechanisms (29). These IRES elements have been grouped into four distinct classes based on their mode of translation initiation (29). Members of classes I and II require all host translation initiation factors except for eIF4E, and class II IRES initiate translation at a start codon without ribosomal scanning. Class III IRES only depend on eIF2, eIF3, and eIF5 for translation, whereas class IV IRES initiate translation in the absence of any eIFs. Previous results showed that QL47 inhibited translation driven by the EMCV IRES element (class II) (6) (Fig. 2*A*). We examined the effects of QL47 on translation driven by representative IRES elements from each class in cells (Fig. 4*C*) and *in vitro* (Fig. 4*D*). Consistent with our prior observation that QL47 inhibits poliovirus and hepatitis C virus (HCV) (6, 9), we observed that the compound inhibits translation driven by the poliovirus and HCV IRES elements (class I and III, respectively). Notably, QL47 also inhibited reporter expression driven by the cricket paralysis virus (CrPV) IRES (class IV), which binds directly to the 40S ribosomal subunit and initiates translation in the absence of other host initiation factors (30) (Fig. 4, *C* and *D*). This indicates that QL47 is unlikely to act by targeting a translation initiation factor directly.

Intrigued by the finding that QL47 blocks protein synthesis without bias for any given initiation mechanism, we decided to evaluate its effect on translation driven by preformed translation complexes by allowing formation of initiation complexes in the *in vitro* translation reactions for 10 min prior to addition of compounds. As expected, the translation elongation inhibitors cycloheximide (CHX) and lactimidomycin (LTM) (3, 31) efficiently blocked translation of the reporter RNA regardless of when they were added to the reaction, whereas harringtonine, an inhibitor of translation initiation (20–22), lost most of its inhibitory effect when added to preformed initiation complexes (Fig. 4*E*). Interestingly, QL47 more closely resembles CHX and LTM in its inhibition of *in vitro* translation from preformed initiation complexes in these experiments. Together, these data indicate that QL47 inhibits protein synthesis with a mechanism of action that is distinct from classical translation initiation inhibitors.

## Discussion

The data we present here suggest that QL47, a broad-spectrum antiviral (6), acts by blocking eukaryotic host translation. Most viruses are absolutely dependent on the host translational machinery, and small-molecule inhibitors of translation have been shown previously to display broad-spectrum antiviral activity (4, 5). In general, targeting a host function rather than a viral target is expected to be advantageous, as it could limit the development of antiviral resistance; however, it is challenging to develop drugs that inhibit such a target without exhibiting unwanted harmful effects to the host cell (32). Although QL47 is a general inhibitor of eukaryotic protein synthesis, it exhibits a significant therapeutic index because antiviral activity can be achieved at concentrations that do not block host cell proliferation (6). Such observations were also made for well-characterized and highly specific small-molecule inhibitors of translation such as LTM (4), homoharringtonine (5), hippuristanol (26), and silvestrol (33), which also display antiviral activity at noncytotoxic concentrations.

One enticing explanation for this is that positive-strand RNA viruses, such as those targeted by QL47, require their genome to be translated as soon as it is delivered to cells. Consequently, treatment with limited concentrations of compounds would lead to temporary blockade of translation at a stage that is critical for establishment of the viral infectious cycle. Accordingly, we observed that inhibition of host protein synthesis was lost when low-concentration treatments of QL47 were applied for 24 h (Fig. 1*E*). Another explanation for this phenomenon is that host cells employ multiple strategies to translate their mRNA, whereas viruses have often evolved specific strategies that rely on hijacking a specific host factor (34). This renders the virus critically dependent on the availability and function of that factor to translate the viral genome (33, 35–37). It is thus an attractive strategy to specifically inhibit viral translation by targeting a viral RNA element, such as a viral IRES (38), or a host protein essential for viral translation but functionally redundant for the host cell. Successful examples of this approach can be found in compounds that selectively target eIF4A, an RNA helicase required to unwind the complex RNA structures in the 5′ UTR of mRNA and of multiple viral RNAs. Despite having a minor effect on host protein biosynthesis, these small molecules greatly impair the establishment of infection for several RNA viruses (26, 33). Although the molecular target of QL47 is not known, it is likely that this compound targets a host factor involved in translation of a specific subset of mRNAs, including viral RNAs.

Aside from its associated antiviral effect, QL47's regulation of translation could have a broader therapeutic effect, in particular against fast-growing tumor cells. In fact, QL47 was first discovered as an anticancer agent (8). Although it has been shown that QL47 inhibition of BTK is primarily responsible for this phenotype (8), our findings described here suggest that the antiproliferative activities of QL47 in B cell lymphoma may additionally be mediated through its effect on translation.

Separate from its therapeutic potential, QL47 is also useful as a probe for studying cellular translation. Historically, small molecules that regulate translation have served as powerful tools to elucidate the function of the prokaryotic ribosome (39). Far fewer eukaryote-specific ribosomal inhibitors have been identified, and we have a limited understanding of their molecular mechanisms (31). From this perspective, QL47 may emerge as a useful tool compound, and elucidation of QL47's mechanism of action and selectivity and specificity profiles is likely to advance our understanding of how eukaryotic translation occurs and is regulated. Toward this goal, we are currently pursuing multiple approaches to identify QL47's cellular target(s). Our observation that QL47 only inhibits eukaryotic translation indicates that the factor is not conserved in prokaryotic cells. Moreover, our experiments performed in rabbit reticulocyte and yeast lysates suggest that the relevant QL47 target is present in both systems. We are currently working toward developing strategies to isolate the target from these lysates.

Identifying the molecular target(s) of QL47 will also help us to further dissect the mechanism of action of this compound. In this work, we showed that QL47 inhibited protein synthesis in a manner strikingly similar to LTM and seemed to block translation at a step downstream of the formation of initiation complexes (Fig. 4, *C–E*) while not preventing translocation of ribosomes on the RNA template, as demonstrated by the loss of polysomes upon QL47 treatment (Fig. 4*A*). LTM has been shown to directly bind to the tRNA binding site in the 60S ribosome, and it has been demonstrated that binding cannot occur when the site is already occupied by tRNA, which explains LTM's lack of efficacy on elongating ribosomes (3, 31). We are currently investigating whether QL47 also directly associates with ribosomal components. In light of QL47's selective antiviral activity, we are also examining the possibility that it prevents viral infection through additional complementary mechanisms that implicate distinct host factors.

## Materials and methods

### Cells

Huh7 and HEK293T cells were cultured in DMEM supplemented with 10% FBS in a 37 °C incubator with 5% CO_2_ and routinely examined to be free of mycoplasma contamination.

### Compound synthesis and characterization

DMSO, CHX, harringtonine, LTM, G418, WP1130, and puromycin were purchased from Millipore/Sigma (D8418 and C4859), Toronto Research Chemicals (H105285), Calbiochem (5.06291.0001), Teknova (G5005), Selleckchem (S2243), and Thermo Fisher Scientific (A1113803), respectively. QL47 (also known as QL-XII-47), QL47R (also known as QL-XII-47R), YKL-04-085, and YKL-03-109 (also known as compound 14) were synthesized using methods reported previously (6, 8, 9).

### Plasmids

Plasmids encoding reporter RNA were as follows: pDENrep-FH encoding a DV replicon that expresses firefly luciferase (40); pcDNA3-RLUC-POLIRES-FLUC, a bicistronic reporter plasmid that expresses firefly luciferase under control of the poliovirus IRES (41); pBS-EMCV-Fluci, which expresses firefly luciferase under control of the EMCV IRES (42); pSGR-JFH1/Luc encoding a HCV replicon that expresses firefly luciferase (43); and VSV-LUC, encoding a recombinant VSV that expresses firefly luciferase (44). The NlucP reporter DENV2 plasmid (pD2repNlucP) was constructed by replacing the firefly luciferase in pDENrep-FH (40) with nanoluciferase fused with a proline, glutamate, serine, threonine domain (NlucP) (17). The pDENrep-FH vector was amplified by PCR using primers 5′-CTACTAAAGCTTGCTGGTGACGTTG-3′ and 5′-GGACACGCGGTTTCTCTCGC-3′. The NlucP insert was amplified by PCR from pNL1.2[NlucP] (Promega, N1011) using primers 5′-GCGAGAGAAACCGCGTGTCCATGGTCTTCACACTCGAAGATTTC-3′ and NlucP_rev 5′-TCACCAGCAAGCTTTAGTAGGACGTTGATGCGAGCTGAAG-3′. The vector and insert fragments were purified on gel, joined by Gibson assembly (New England Biolabs, E2611), and transformed in ElectroMAX^TM^ Stbl4^TM^–competent cells (Thermo Fisher Scientific, 11635018).

The bicistronic reporter plasmid pcDNA3-RLUC-CrPV-IRES-FLUC was constructed by exchanging the poliovirus IRES from pcDNA3-RLUC-POLIRES-FLUC with that of CrPV from pFR-CrPV (45). DNA fragments were amplified by PCR using primer pairs 5′-ccaagcttgggctgcaggtcAAAGCAAAAATGTGATCTTG-3′/5′-tttatgtttttggcgtcttcGGTGGCTAGATTATCTTG-3′ and 5′-GAAGACGCCAAAAACATAAAG-3′/5′-GACCTGCAGCCCAAGCTTG-3′, and the plasmid was assembled using NEBuilder® HiFi DNA Assembly Master Mix (New England Biolabs, E2621) according to the manufacturer's instructions. Plasmids were sequenced at the Dana-Farber Harvard Cancer Center DNA sequencing facility.

### In vitro transcription

*In vitro* transcripts were synthesized from *Pst*I-linearized, Klenow-treated pDENrep-FH and *Cla*I-linearized pD2repNlucP using the AmpliScribe T7-Flash transcription kit (Lucigen, ASF3257) and m7G(5′)ppp(5′)A RNA cap structure analog (New England Biolabs, S1405) according to the manufacturers' instructions. For pcDNA3-RLUC-POLIRES-FLUC and pcDNA3-RLUC-CrPV-IRES-FLUC, *in vitro* transcripts were synthesized from *Xho*I-linearized plasmids using the mMessage mMachine T7 transcription kit (Thermo Fisher Scientific, AM1344) and then polyadenylated using *E. coli* poly(A) polymerase (New England Biolabs, M0276). *In vitro* transcripts were synthesized from *Xho*I-linearized pBS-EMCV-Fluci and *Xba*I-linearized mung bean nuclease–treated pSGR-JFH1/Luc using the AmpliScribe T7-Flash transcription kit (Lucigen, ASF3257).

Firefly luciferase reporter mRNA bearing the conserved VSV gene start and end sequences was transcribed *in vitro* using 10 μg of detergent-activated rVSV-FLUC (44) as described previously (46, 47). Briefly, rVSV-FLUC virions were incubated for 5 h at 30 °C in a buffer consisting of 30 mm Tris HCl (pH 8.0), 33 mm NH_4_Cl, 10 mm KCl, 4.5 mm Mg(OAc)_2_, 10 mm EGTA, 1 mm DL-DTT, 0.2 mm spermidine, and 0.05% Triton X-100. The reaction was supplemented with 1 mm SAM, 1 mm ATP, CTP/GTP/UTP (0.5 mm each), 0.05 mg/ml actinomycin D-mannitol (Sigma-Aldrich, A5156), murine RNase inhibitor (New England BioLabs, M0314), and 30% (v/v) rabbit reticulocyte lysate (Promega, L4960). RNA was isolated by column purification using the RNeasy Mini Kit (Qiagen, 74104) following the manufacturer's protocol and eluted in water.

### Antibodies

Mouse anti-puromycin antibody (PMY-2A4) was obtained from Developmental Studies Hybridoma Bank and deposited to the Developmental Studies Hybridoma Bank by Jonathan Yewdell. Rabbit mAb against tubulin, rabbit polyclonal antibody against GFP, and mouse mAb against the His_6_ tag were purchased from Cell Signaling Technology (2125), Millipore/Sigma (G1544), and GenScript (A00186), respectively. HRP-conjugated goat anti-mouse IgG and anti-rabbit IgG antibodies were obtained from Bio-Rad (170-6516 and 170-6515, respectively). IRDye® 800CW–conjugated goat anti-rabbit IgG antibody and IRDye® 680LT-conjugated goat anti-mouse IgG antibody were purchased from LI-COR (926-32211 and 926-68020, respectively).

### Reporter assays in live cells

HEK293T cells were seeded in DMEM supplemented with 10% FBS in a 96-well plate. After 24 h, the medium was changed to FreeStyle^TM^ 293 expression medium (Thermo Fisher Scientific, 12338018) supplemented with 10 mm HEPES and Nano-Glo® live-cell substrate mix (Promega, N2011) according to the manufacturer's instructions. Cells were immediately transfected with 50 ng of *in vitro* transcribed RNA using Lipofectamine MessengerMAX transfection reagent (Thermo Fisher Scientific, LMRNA003). Luminescence was then recorded every minute for a total of 4 h in a Synergy plate reader (BioTek) with the temperature control set at 37 °C. Compounds were added to triplicate wells 1 h after the start of the experiment.

For assays performed in live bacteria, we used the *E. coli* K-12 W3110 strain carrying the pUA66-rrnB plasmid that expresses GFP under control of the constitutive rrnB ribosomal promoter, specifying strong and constitutive fluorescence (24). Saturated starter cultures were diluted 1:100 in fresh Luria-Bertani medium supplemented with kanamycin at 50 μg/ml and grown for 1 h at 37 °C. The culture was next diluted 1:100 in fresh medium and distributed in a clear 96-well plate at 100 μl per well. Small molecules were diluted in fresh medium at the indicated concentrations and added to wells containing the bacterial culture at 100 μl per well. The plate was next incubated at 37 °C for 14 h, and GFP fluorescence (excitation at 485 nm and emission at 528 nm) was measured every 10 min after a shaking step using a Synergy plate reader (BioTek).

### Cellular reporter assays

Huh7 cells seeded in a 48-well plate were treated with small molecules and immediately transfected with *in vitro* transcripts using Lipofectamine MessengerMAX transfection reagent (Thermo Fisher Scientific, LMRNA003). Cells were lysed and processed according to the instructions for the luciferase assay system (Promega, E1501), and the luciferase signal was measured using a Synergy plate reader (BioTek).

### In vitro translation assays

For assays using rabbit reticulocyte lysates, the indicated *in vitro* transcript (100 ng) was added to 12 μl of translation reactions assembled according to the manufacturer's instructions (Promega, L4540). Reactions were incubated with small molecules at 30 °C for the indicated time, and 5-μl aliquots were assayed for expression of firefly luciferase according to the instructions in the luciferase assay system (Promega, E1501). The luciferase signal was measured using a Synergy plate reader (BioTek).

Reactions using the FluoroTect^TM^ Green_Lys_ *in vitro* translation labeling system (Promega, L5001) were performed using the Flexi® rabbit reticulocyte lysate system (Promega, L4540) according to the manufacturer's instructions. Translation reactions were assembled in 12 μl and incubated at 30 °C for 90 min in the presence of small molecules. 5-μl aliquots were fractionated on a 10% polyacrylamide gel (Bio-Rad, 4561033), and fluorescence (excitation at 495 nm and emission at 519 nm) was measured using a Typhoon FLA 9500 (GE Healthcare Life Sciences). Band intensities were quantified using ImageJ software.

Translation reactions in a reconstituted *E. coli* cell-free synthesis system (PURExpress®) were performed according to the manufacturer's instructions (New England Biolabs, 6840). A plasmid expressing GFP under control of a T7 promoter was used as a template, and translation reactions were assembled in 12 μl and incubated at 37 °C for 1 h in the presence of small molecules. 5-μl aliquots were analyzed by Western blotting using GFP and histidine tag antibodies.

Yeast translation extracts were prepared from *Saccharomyces cerevisiae* in logarithmic growth phase as described previously with some modifications (48–50). Cells were washed four times in buffer A (30 mm HEPES (pH 7.6), 100 mm Mg(OAc)_2_, and 2 mm K(OAc)) supplemented with 8.5% mannitol (w/v). Cells were lysed by manual glass bead lysis using 1.5 ml per gram of wet weight buffer A supplemented with 8.5% mannitol and 0.5 mm PMSF. Lysates were clarified twice to generate an “S30 lysate” that was passed through a Zeba Desalt Spin Column (Thermo Fisher Scientific, 89894) pre-equilibrated with buffer A plus 8.5% mannitol and 0.5 mm PMSF. Extracts were stored at −80 °C until use. *In vitro* translation using rVSV-FLUC–transcribed mRNA was performed as described previously (36). Prior to use for *in vitro* translation, endogenous yeast mRNAs were digested by treating lysates with 0.7 mm CaCl_2_ and micrococcal nuclease (New England Biolabs, M0247) for 10 min at 25 °C. The reaction was stopped by adding 2.3 mm EGTA. Translation reaction mixtures consisted of 50% (v/v) yeast lysate and a final concentration of 0.84 mm ATP, 0.21 mm GTP, 21 mm creatine phosphate (Sigma, 10621714001), 45 units/ml creatine phosphokinase (Sigma, 10127566001), 2 mm DTT, 2.5 mm Mg(OAc)_2_, 100 mm K(OAc), 8 μm amino acids (Promega, L4461), 255 μm spermidine, and 1000 units/ml murine RNase inhibitor. Small molecules were added to the reaction mixtures on ice, and *in vitro* transcribed reporter RNA was added last. Reactions were incubated at 25 °C for 2 h, and luciferase expression was assayed in a SpectraMax L microplate reader.

### Metabolic labeling

For the puromycin incorporation assay, Huh7 cells were seeded in 24-well plates and treated with small molecules for 1 h before adding puromycin (1 μm) for 30 min. Cells were washed with PBS and lysed by adding radioimmune precipitation assay buffer (50 mm Tris-HCl, 150 mm NaCl, 1% NP-40, 0.5% sodium deoxycholate, and 0.1% SDS (pH 7.4 ± 0.2)) with protease inhibitor (Roche), PhosSTOP phosphatase inhibitor (Roche, 4906845001), and 2.5 units/ml universal nuclease for cell lysis (Pierce, 88700). The lysates were clarified by centrifugation at 21,000 × *g* for 30 min at 4 °C, and the concentration of the lysate was determined using the BCA protocol (Pierce). Equal amounts of proteins were analyzed by Western blotting using anti-tubulin and anti-puromycin antibodies.

For metabolic labeling of proteins using radiolabeled amino acids, Huh7 cells were seeded in 24-well plates and treated with small molecules at the time indicated for each experiment. At the time indicated for labeling of neosynthesized proteins, cells were washed with PBS, and DMEM without cysteine and methionine (Corning, 17-204-CI) supplemented with l-glutamine (Thermo Fisher Scientific 25030-081) and 2% FBS was then added to the cells. Cells were incubated for 30 min at 37 °C to deplete cellular amino acid pools. 14 μCi of EasyTag^TM^ Express ^35^S Protein Labeling Mix (PerkinElmer Life Sciences, NEG7720) was then added to each well, and cells were incubated for 30 min at 37 °C. Cells were washed with PBS and lysed in radioimmune precipitation assay buffer (Boston BioProducts, BP-419) on ice for 10 min. Equal volumes of cell lysates were fractionated on a 10% polyacrylamide gel (Bio-Rad, 4561033), and the gel was dried using the DryEase^TM^ Mini-Gel Drying System (Thermo Fisher Scientific, NI2387). The signal was visualized using a storage phosphorimaging system (GE Healthcare Life Sciences). Band intensities were quantified using ImageJ software.

### Western blotting

Equal amounts of proteins were fractionated on 4%–20% polyacrylamide gels (GenScript, M42015) or 4%–12% BisTris polyacrylamide gels (Life Technologies, NW04122BOX) and transferred to a polyvinylidene difluoride membrane (Millipore/Sigma, IPVH00010) or nitrocellulose membrane (Bio-Rad, 1620112). When using HRP-conjugated antibodies, the membrane was blocked for 1 h at room temperature in PBS-Tween 20 (PBS-T) plus 5% (w/v) milk and then incubated overnight at 4 °C with appropriate dilutions of the primary antibodies. The membrane was then washed in PBS-T, followed by incubation for 1 h at room temperature in the presence of HRP-conjugated secondary antibodies (1:3000 dilution). After washes in PBS-T, the membrane was developed with enhanced chemiluminescence reagents (Thermo Fisher Scientific, PI32106), and the signal was captured using the Amersham Biosciences Imager 600 (GE Healthcare).

When using LI-COR reagents, membranes were blocked with LI-COR blocking solution and incubated with primary antibodies overnight, followed by three washes in LI-COR blocking solution and incubation with secondary antibodies (1:10,000 dilution) for 1 h in the dark. After three final washes, the membranes were imaged on a LI-COR fluorescent imaging station. Band intensities were quantified using ImageJ software.

### Polysome profiling

Huh7 cells were seeded in 75-cm^2^ flasks and treated with the indicated small molecules for 3 h at 37 °C. Cells were washed with cold PBS supplemented with 0.1 mg/ml cycloheximide and collected by cell scraping in 1 ml of cold PBS supplemented with 0.1 mg/ml cycloheximide. The cells were spun for 5 min at 1,000 × *g* at 4 °C. The cell pellet was resuspended in 5 mm Tris (pH 7.4), 2.5 mm MgCl_2_, 1.5 mm KCl, 3.3 μm DTT, 0.5% (v/v) Triton-X100, and 0.5% (v/v) sodium deoxycholate, vortexed briefly, and incubated on ice for 15 min. The lysate was spun for 2 min at 4 °C at 10,000 × *g*, and the supernatant was resolved on a continuous 10%–45% (w/v) sucrose gradient prepared in 15 mm Tris (pH 7.4), 15 mm MgCl_2_, and 150 mm NaCl by centrifugation at 40,000 rpm at 4 °C for 2 h in a Beckman SW40 Ti rotor. Fractions were collected from the top of the gradient using a Biocomp fractionator and analyzed with an in-line spectrophotometer (Bio-Rad) measuring optical density at 260 nm. GraphPad Prism was used to calculate the area under the curve for each peak.

### Statistical analyses

An unpaired t test was used to compare quantitative data. GraphPad Prism was used for all statistical analyses.

## Author contributions

M. d. W., M. C., C. M. O., N. S. G., and P. L. Y. conceptualization; M. d. W., M. C., D. J. B., W. J. N., C. M. O., I. R., Y. L., N. S. G., and P. L. Y. formal analysis; M. d. W., J. W., S. P. J. W., N. S. G., and P. L. Y. supervision; M. d. W., M. C., D. J. B., W. J. N., C. M. O., I. R., and Y. L. investigation; M. d. W. visualization; M. d. W., N. S. G., and P. L. Y. writing-original draft; M. d. W., M. C., D. J. B., W. J. N., C. M. O., I. R., N. S. G., and P. L. Y. writing-review and editing; J. W. project administration; S. P. J. W., N. S. G., and P. L. Y. funding acquisition.

## Supplementary Material

Supporting Information

## Footnotes

4 The abbreviations used are:

DV: dengue virus
BTK: Bruton's tyrosine kinase
SAR: structure–activity relationship
EMCV: encephalomyocarditis virus
IRES: internal ribosomal entry site(s)
HCV: hepatitis C virus
CrPV: cricket paralysis virus
CHX: cycloheximide
LTM: lactimidomycin
VSV: vesicular stomatitis virus
PV: poliovirus.
