# Supplementary material for: A broad-spectrum antiviral molecule, QL47, selectively inhibits eukaryotic translation
Source: J Biol Chem. 2019 Dec 30;295(6):1694–703. doi: 10.1074/jbc.RA119.011132 (PMC7008383; doi:10.1074/jbc.RA119.011132)
Supplement: Supporting Information [file supp_295_6_1694__index.html]

A broad-spectrum antiviral molecule, QL47, selectively inhibits eukaryotic translation — Small molecule inhibition of eukaryotic translation — A broad-spectrum antiviral molecule, QL47, selectively inhibits eukaryotic translation — QL47 selectively inhibits eukaryotic translation — Supporting Information 

# A broad-spectrum antiviral molecule, QL47, selectively inhibits eukaryotic translation

## Supporting Information

- Supporting Information (to be published online) - Supporting information
