# Supplementary material for: A broad-spectrum antiviral molecule, QL47, selectively inhibits eukaryotic translation
Source: J Biol Chem. 2019 Dec 30;295(6):1694–703. doi: 10.1074/jbc.RA119.011132 (PMC7008383; doi:10.1074/jbc.RA119.011132)
Supplement: Supporting Information [file supp_RA119.011132_155932_1_supp_444611_q2lq61.pdf]

Supporting Information

A broad-spectrum antiviral molecule, QL47, selectively inhibits eukaryotic translation

Mélissanne de Wispelaere[1#], Margot Carocci[1#], Dominique J. Burri[1‡], William J. Neidermyer Jr.[1‡], Calla M. Olson[2,3‡], Imme Roggenbach[1‡], Yanke Liang[2,3], Jinhua Wang[2,3], Sean P. J. Whelan[1], Nathanael S. Gray[2,3], Priscilla L. Yang[1*]

Affiliations:

[1] Department of Microbiology and Blavatnik Institute, Harvard Medical School, Boston, Massachusetts, USA

[2] Department of Biological Chemistry and Molecular Pharmacology, Harvard Medical School, Boston, Massachusetts, USA

[3] Department of Cancer Biology, Dana-Farber Cancer Institute, Boston, Massachusetts, USA

[#,‡] Contributed equally

* Lead Contact and corresponding author. Email: priscilla_yang@hms.harvard.edu

List of materials included

Figures

- **Figure S1**
- **Figure S2**

Supplementary methods

Supplementary references

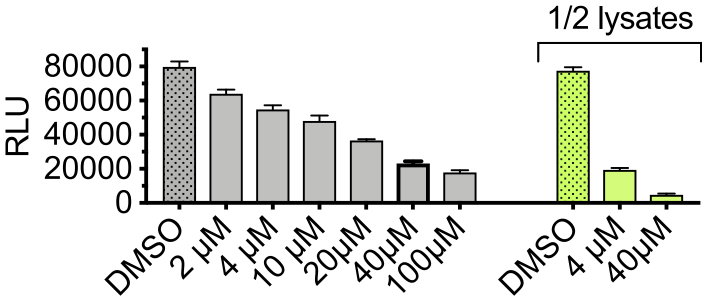

Figure S1: Translation of *in vitro*-transcribed reporter DV subgenomic RNA in rabbit reticulocyte lysates was performed for 90 minutes at 30 °C in the presence of DMSO or the indicated concentrations of QL47. Where indicated, the translation reactions were performed using half of the rabbit reticulocyte lysates. The luciferase signal was measured, and data are presented as means ± standard deviation of n = 4 technical replicates. One representative experiment is shown from n = 2 independent experiments.

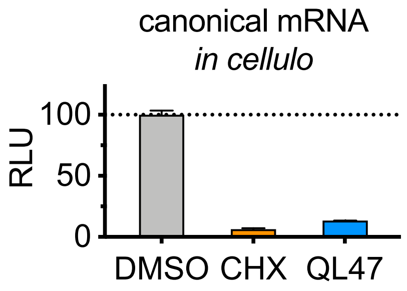

Figure S2: Analysis of translation assays performed in live cells. Huh7 cells were transfected with an *in vitro*-transcribed reporter RNA harboring a m7G(5')ppp(5')G cap structure and a poly(A) tail. Cells were immediately treated with DMSO, 30 μg/mL cycloheximide (CHX), or 2 μM QL47. Intracellular luciferase signal corresponding to canonical cap-dependent translation of the reporter luciferase protein was measured at 6 hours post-treatment and data are presented as means normalized to DMSO ± standard deviation of n = 2 experimental replicates. One representative experiment is shown from n = 3 independent experiments.

Supplementary methods

In vitro transcription of reporter mRNA

In vitro transcripts were synthesized from *Xho*I-linearized pBS-Rluci (1) using the mMessage mMachine T7 transcription kit (ThermoFisher Scientific, AM1344), then polyadenylated using *E. coli* poly(A) polymerase (New England BioLabs, M0276).

Cellular reporter assays

Huh7 cells seeded in a 48-well plate were treated with small molecules and immediately transfected with *in vitro* transcripts using the Lipofectamine MessengerMAX transfection reagent (ThermoFisher Scientific, LMRNA003). Cell were lysed and processed according to the instructions in the *Renilla* luciferase assay system (Promega, E2820) and luciferase signal was measured using a Synergy plate reader (BioTek).

Supplementary references

1. Murakami, K., Kimura, T., Osaki, M., Ishii, K., Miyamura, T., Suzuki, T., Wakita, T., and Shoji, I. (2008) Virological characterization of the hepatitis C virus JFH-1 strain in lymphocytic cell lines. *J. Gen. Virol.* **89**, 1587–1592
